# Dr. Manuel Amador Guerrero

**Published:** 1909-05-15

**Authors:** 


### OBITUARY.

The death is announced, at the age of seventy-five, of Dr. Manuel Amador Guerrero, a former President of the Republic of Panama. Dr. Guerrero was a physician, and he had the especial honour of being elected first President of the Republic shortly after its declaration of independence at the close of the year 1903. He held the post of President for the statutory term of four years. Dr. Guerrero visited Europe in 1907, but did not come to London.

